# Comparative Efficacy of Er,Cr:YSGG Laser, Electrolytic Cleaning, and Chlorhexidine for the Decontamination of Sandblasted, Large-Grit, Acid-Etched (SLA) Implant Surfaces: An In Vitro Controlled Study

**DOI:** 10.7759/cureus.113294

**Published:** 2026-07-24

**Authors:** Charbel Chedid, Karim Corbani, Rim Bourgi, Emile Chrabieh

**Affiliations:** 1 Faculty of Dental Medicine, Saint Joseph University of Beirut, Beirut, LBN; 2 Department of Restorative and Esthetic Dentistry, Faculty of Dental Medicine, Saint Joseph University of Beirut, Beirut, LBN; 3 Laser Unit, Faculty of Dental Medicine, Saint Joseph University of Beirut, Beirut, LBN; 4 Craniofacial Research Laboratory, Faculty of Dental Medicine, Saint Joseph University of Beirut, Beirut, LBN; 5 Department of Oral Surgery and Implantology, Faculty of Dental Medicine, Saint Joseph University of Beirut, Beirut, LBN

**Keywords:** biofilms, dental implants, electrolysis, lasers, peri-implantitis

## Abstract

Background: Predictable decontamination of exposed implant surfaces remains difficult to achieve in peri-implantitis therapy. Rough sandblasted, large-grit, acid-etched (SLA) surfaces and implant thread geometry may promote biofilm retention and limit the effectiveness of chemical antiseptics and mechanical instrumentation.

Objective: This controlled in vitro study compared the antimicrobial efficacy of three clinically relevant decontamination approaches: 0.12% chlorhexidine (CHX), GalvoSurge electrolytic cleaning, and erbium, chromium: yttrium-scandium-gallium-garnet (Er,Cr:YSGG) laser irradiation against *Staphylococcus aureus* biofilms formed on Straumann SLA tissue-level implants in a standardized peri-implant defect model.

Methods: Forty-eight Straumann SLA tissue-level implants were fixed in custom three-dimensional (3D)-printed peri-implant defect models and individually contaminated with *Staphylococcus aureus* and incubated in brain-heart infusion broth for 72 hours at 37°C. Implants were randomly assigned to four groups (n = 12/group): untreated control, CHX immersion for 60 s, GalvoSurge electrolytic cleaning for 120 s, or Er,Cr:YSGG laser treatment (2780 nm; 2.5 W; 30 Hz; 70% water; 60% air; 30 s). Residual viable bacteria were recovered using a standardized microbrush protocol and expressed as log10(colony-forming units (CFU)/implant).

Results: Mean log10(CFU/implant) values were highest in the untreated control group (5.09 ± 0.33), followed by CHX (4.03 ± 0.12), GalvoSurge (3.77 ± 0.25), and Er,Cr:YSGG laser treatment (2.52 ± 0.39). The corresponding reductions relative to control were approximately 1.06 log10 for CHX, 1.32 log10 for GalvoSurge, and 2.57 log10 for laser treatment. Statistical analysis demonstrated a significant treatment effect (p < 0.001), with laser irradiation achieving the greatest reduction in viable bacterial counts under the tested conditions.

Conclusion: All active protocols reduced viable *Staphylococcus aureus* counts compared with untreated implants. Within the limitations of this single-species in vitro model, Er,Cr:YSGG laser irradiation achieved the greatest reduction, whereas GalvoSurge and CHX resulted in moderate and comparable decreases. Further studies incorporating multispecies biofilms, assessment of implant surface integrity, and clinically relevant outcomes are warranted.

## Introduction

Dental implants represent a well-established and highly predictable treatment modality for restoring esthetic, functional, and phonetic deficiencies within the oral cavity. In implant therapy, early success is primarily determined by the achievement of primary stability, followed by secondary osseointegration after placement within the jawbone [1-3]. Long-term success, however, relies on the preservation of sufficient peri-implant bone volume and quality, ensuring the ability to withstand functional loads transmitted through the prosthetic restoration [1].

Adequate bone availability at the intended implant site is a critical prerequisite for both immediate and long-term treatment outcomes [3]. For optimal osseointegration, the implant surface must be completely surrounded by bone. Any deficiency in bone-to-implant contact may impair early integration and increase the risk of long-term failure [2].

Following successful osseointegration, biofilm-associated peri-implant diseases represent one of the most common biological complications. Clinically, these conditions are characterized by bleeding on probing and/or suppuration, accompanied by progressive bone loss [4]. Their prevalence varies depending on diagnostic criteria, follow-up duration, and patient-related risk factors [3,4].

This clinical challenge is closely linked to implant design. Moderately rough titanium surfaces, such as sandblasted, large-grit, acid-etched (SLA) surfaces, improve osseointegration but may also favor plaque retention once exposed to the oral environment [5-7]. In peri-implant defects, implant threads, undercuts, and limited access areas can shelter bacterial biofilm from mechanical instruments, antiseptics, and irrigating solutions, leading to residual contamination even after thorough debridement [8,9].

Conventional mechanical debridement techniques, including hand instruments, ultrasonic devices, and air-abrasive systems, can reduce biofilm load but often fail to achieve complete decontamination. These approaches may also induce minor alterations to the implant surface [5,10]. Consequently, alternative or adjunctive decontamination strategies have been investigated.

Chemical antiseptics, laser-based therapies, and electrochemical approaches have been proposed for implant surface decontamination; however, no universally accepted protocol exists [10-12]. Among emerging technologies, electrolytic cleaning systems have been introduced as novel approaches aiming to disrupt biofilm through electrochemical reactions and potentially enhance surface wettability, which may improve decontamination efficacy [6-8]. Given these limitations, well-controlled experimental models using standardized implants, contamination protocols, and outcome assessments are needed to better compare the antimicrobial performance of different decontamination strategies. Therefore, the comparison of these different treatment modalities is clinically relevant, as they represent distinct mechanisms currently investigated for managing biofilm contamination on implant surfaces.

The efficacy of decontamination strategies is commonly assessed by quantifying bacterial reduction, typically expressed as colony-forming units (CFU), with results often reported on a logarithmic scale (log_10_CFU) to allow standardized comparison of microbial load [13-15].

Therefore, the aim of this in vitro study was to evaluate and compare the antimicrobial efficacy of different peri-implant decontamination modalities, including 0.12% chlorhexidine (CHX), GalvoSurge electrolytic cleaning, and erbium, chromium: yttrium-scandium-gallium-garnet (Er,Cr:YSGG) laser irradiation, against *Staphylococcus aureus* biofilm formed on Straumann SLA tissue-level implants mounted in a custom peri-implant defect model. The primary outcome was bacterial reduction expressed as log_10_(CFU/implant), whereas the standardized implant model and biofilm contamination protocol were used as methodological approaches to ensure controlled comparison between treatment groups. The null hypothesis was that no significant difference in log_10_(CFU/implant) would be observed among the untreated control, CHX, GalvoSurge, and laser treatment groups.

## Materials and methods

Ethics committee approval 

As stated in the letter dated September 19, 2025, issued by the Department of Oral Implantology, Faculty of Dentistry, Saint-Joseph University of Beirut, this study (file no. Tfemd-2026-18) was reviewed at the meeting of the Saint-Joseph University of Beirut, Faculty of Dentistry Clinical Research Ethics Committee and was found ethically appropriate.

Determination of sample size

This was an in vitro controlled comparative study evaluating four implant surface conditions: untreated control, 0.12% CHX decontamination, GalvoSurge® (Straumann, Basel, Switzerland), and Er,Cr:YSGG laser solid-state. 

A total of 48 SLA tissue-level titanium dental implants (Straumann® SP WN SLA, 4.8 mm × 10 mm) were included (n = 12/group). The sample size of 12 implants per group was selected based on the expected large effects reported in previous in vitro implant decontamination studies using CFU outcomes [11,16,17]. In controlled microbiological models, variability is generally limited, and treatment effects are typically large, allowing detection of biologically relevant differences with moderate group sizes. After data collection, effect-size estimation for the overall treatment effect showed η² = 0.883, indicating that 88.3% of the variance in bacterial load was attributable to treatment at α = 0.05. This value is an effect-size estimate and was not interpreted as statistical power. The primary outcome was viable bacterial load expressed as CFU per implant. All procedures were performed by a single operator in Wakim Laboratory (Jounieh, Lebanon).

Experimental model, biofilm formation, and decontamination procedures

Each implant was mounted in a custom three-dimensional-printed model designed to simulate a standardized peri-implant defect while ensuring mechanical stability during contamination, decontamination, and sampling procedures. The model was designed using computer-aided design (CAD) software and fabricated using additive manufacturing technology. The defect geometry was standardized to expose the coronal implant threads while maintaining complete embedding of the apical portion, thereby simulating the implant surface exposure encountered in peri-implant bone loss. All implants were positioned at the same depth and orientation within the model to ensure identical exposure of the contaminated surface area among specimens. The model covered the apical portion of the implant while leaving the predetermined coronal threads exposed, restricting contact exclusively to the contamination medium, the assigned decontamination protocol, and the sampling instrument.

To standardize the simulated peri-implant defect, the level of implant exposure was predetermined before specimen preparation, with the same number of implant threads exposed in all samples. This approach ensured comparable biofilm accumulation and equivalent accessibility of the contaminated implant surface during decontamination procedures.

Biofilm formation was achieved by placing each implant individually in a sterile container containing 40 mL of brain-heart infusion broth supplemented with 4 mL of *Staphylococcus aureus *inoculum adjusted to 3 McFarland in sterile saline. Samples were incubated at 37°C for 72 hours under controlled conditions to allow biofilm development. All decontamination procedures were performed at room temperature under standardized laboratory conditions. The 72-hour incubation period was selected because in vitro biofilm susceptibility studies have used 72-hour *Staphylococcus aureus* biofilms as older or mature biofilm models for antimicrobial testing, reporting increased cellular density and/or greater antimicrobial tolerance compared with 24-hour biofilms [18,19]. Individual contamination prevented cross-interference and preserved each implant as an independent experimental unit.

Following contamination, implants were randomly assigned to four groups (n = 12 per group): Group 1, Control (no decontamination); Group 2, 0.12% (CHX); Group 3, GalvoSurge; Group 4, Er,Cr:YSGG laser. Decontamination procedures were performed immediately after removal from the contamination medium under standardized environmental conditions to minimize variability.

Implants in the control group did not undergo any decontamination procedure but were handled identically and exposed to ambient conditions for a duration equivalent to the longest treatment time prior to sampling. Chemical decontamination consisted of immersion in 0.12% CHX for 60 seconds, followed by rinsing with sterile saline (0.9% NaCl) for 20 seconds to neutralize residual antimicrobial activity and prevent carryover effects during microbiological analysis. Electrolytic cleaning was carried out using the GalvoSurge system (Straumann AG, Basel, Switzerland) according to the manufacturer's clinical workflow. The system generates electrochemical reactions at the implant surface through the controlled application of an electrolyte solution and an electric current, resulting in the disruption and removal of biofilm deposits. The applicator was positioned to ensure complete electrolyte contact and coverage of all exposed implant threads. The device was activated for 120 seconds, corresponding to the recommended clinical application time, while maintaining continuous electrolyte flow throughout the procedure. Laser decontamination was performed using a Waterlase MD Er,Cr:YSGG system (BIOLASE, Irvine, CA, USA; 2780 nm) operated in H mode with an MZ6 tip at 2.5 W and 30 Hz, with 70% water and 60% air in non-contact mode. The tip was oriented at 90 degrees relative to the implant surface and applied along the exposed threads for a standardized duration of 30 seconds to ensure complete coverage of the contaminated area. A cohort flowchart illustrating the implant grouping was presented in Table 1.

**Table 1:** Experimental groups and decontamination protocols CHX: chlorhexidine

Group	n	Protocol	Key parameters	Sampling after treatment
Control	12	No decontamination	Handled identically and exposed to ambient conditions for equivalent time	Microbrush recovery into 1 mL sterile saline
CHX	12	Chemical decontamination	0.12% CHX immersion for 60 s; sterile saline rinse 20 s	Microbrush recovery into 1 mL sterile saline
GalvoSurge	12	Electrolytic cleaning	Manufacturer workflow; standardized 120 s activation; complete electrolyte contact with exposed threads	Microbrush recovery into 1 mL sterile saline
Laser	12	Er,Cr:YSGG laser	Waterlase MD (BIOLASE, Irvine, CA, USA); 2780 nm; H mode; MZ6 tip; 2.5 W; 30 Hz; 70% water; 60% air; 30 s	Microbrush recovery into 1 mL sterile saline

Following biofilm formation, implants were randomly allocated to the experimental groups using a computer-generated randomization sequence to ensure balanced distribution among treatment conditions. The operator responsible for biofilm recovery and CFU quantification was blinded to the treatment allocation.

Biofilm recovery and CFU quantification

**Figure 1:**
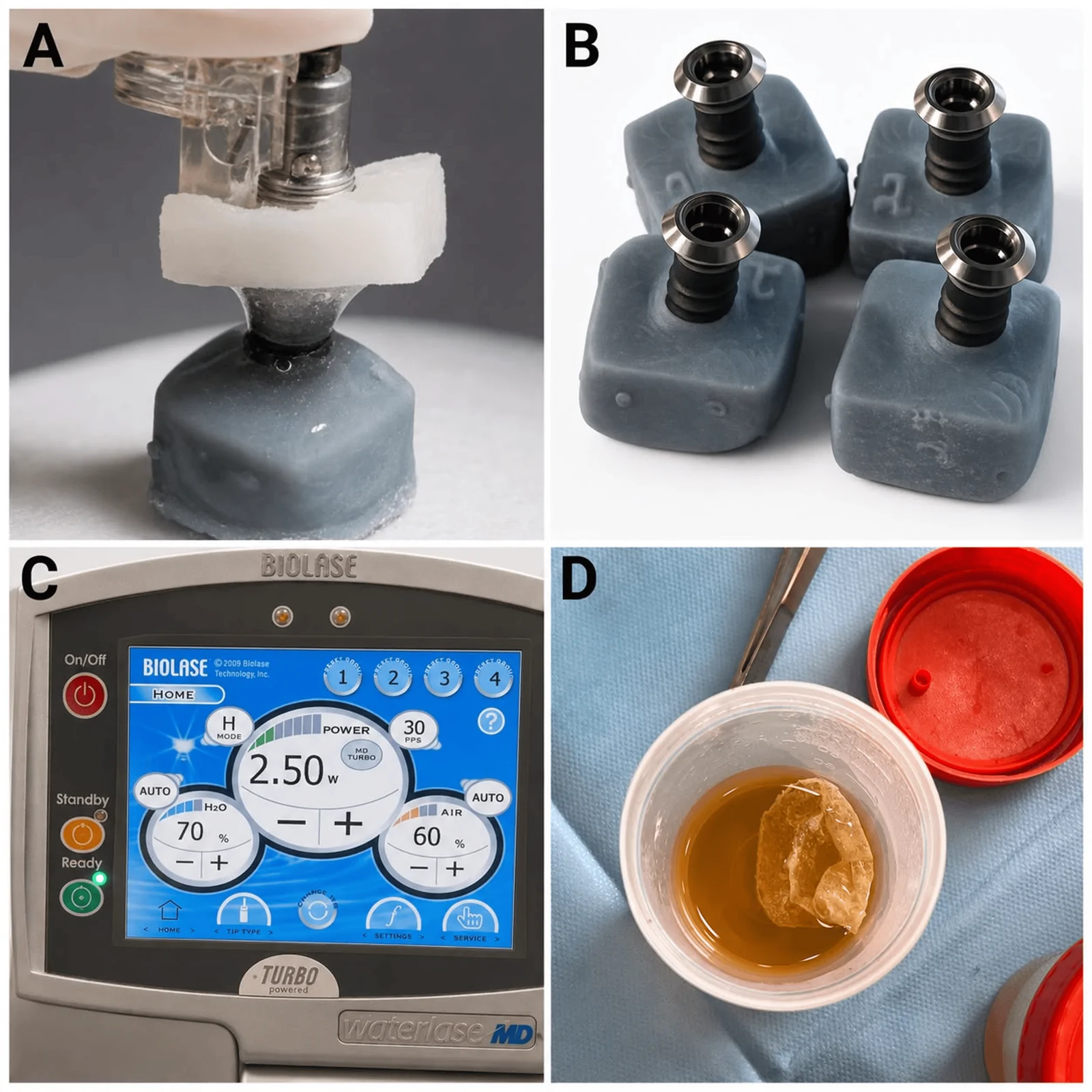
Experimental setup and decontamination protocol (A) Close-up view of the implant specimen during electrolytic cleaning treatment, showing the working tip positioned adjacent to the implant surface. (B) Standardized implant specimens mounted in individual experimental blocks before allocation to the study groups. (C) Er,Cr:YSGG laser unit used for implant surface decontamination, showing the selected operating parameters. (D) Implant specimen immersed in storage medium, showing a macroscopically visible structure suggestive of biofilm maturation

After treatment, residual biofilm was collected using a sterile microbrush passed circumferentially along all exposed implant threads for approximately 15 seconds per implant. The microbrush tip was transferred into 1 mL of sterile saline, vortexed, and subjected to serial dilution. Aliquots of 0.1 mL were plated onto agar media for colony enumeration. CFU per implant was calculated using the standard plate count method, with preference given to plates containing 30 to 300 colonies. CFU values were log_10_-transformed prior to analysis to account for the skewed distribution typical of microbiological data [14,15]. Colony counting was performed by a calibrated examiner using standardized plate-counting criteria. Plates containing 30-300 colonies were considered valid for enumeration, and duplicate readings were performed to ensure counting consistency. Representative images of the experimental setup have been added to enhance methodological transparency and facilitate replication of the experimental protocol (Figure 1).

Statistical analysis

**Figure 2:**
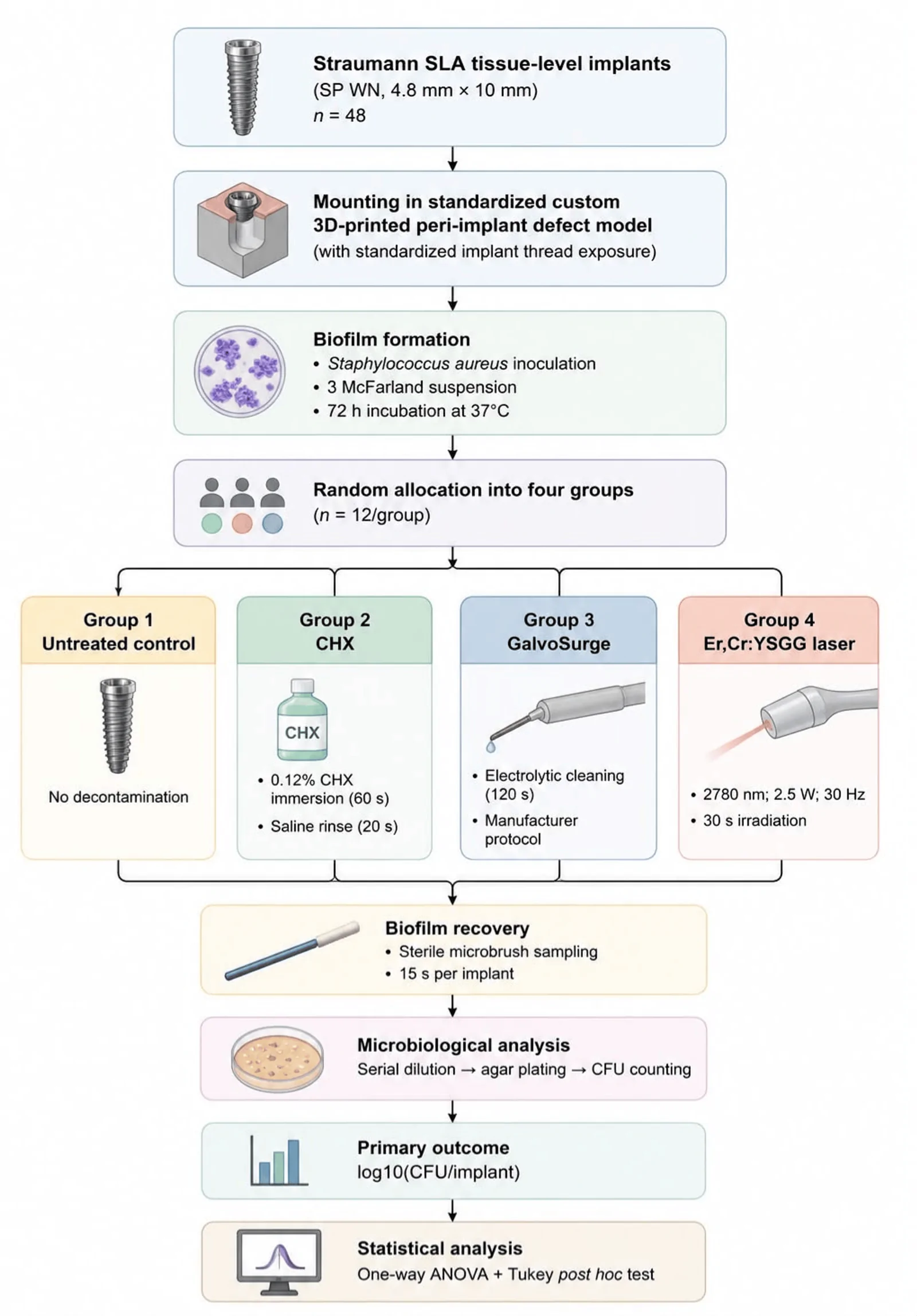
The experimental workflow Image credit: created by the author (RB) using Microsoft PowerPoint (Microsoft Corporation, Redmond, WA, USA)

Statistical analyses were conducted using RStudio (version 2025.09.0, “Cucumberleaf Sunflower” Release; R Foundation for Statistical Computing, Vienna, Austria). One-way analysis of variance (ANOVA) was used for group comparison, with Tukey post hoc testing for pairwise contrasts. When homogeneity of variance was not fully satisfied, Welch robust testing and complementary nonparametric analysis were used to assess the stability of the findings. Statistical significance was set at p < 0.05. Several steps were incorporated to improve reproducibility: the experimental unit was the implant, all groups had equal sample size, decontamination times were standardized, and the same recovery, dilution, plating, and counting workflow was applied across all samples. These details are reported to facilitate replication and comparison with future implant-biofilm studies. A schematic flowchart illustrating the experimental workflow is presented in Figure 2.

## Results

Bacterial recovery and descriptive findings

All 48 implants were included in the analysis, with 12 implants per group. The untreated control group exhibited the highest bacterial load, confirming successful biofilm formation and recovery. Mean log_10_(CFU/implant) values decreased progressively from control (5.09 +/- 0.33) to CHX (4.03 +/- 0.12), GalvoSurge (3.77 +/- 0.25), and laser treatment (2.52 +/- 0.39), indicating a clear reduction gradient across the tested protocols. Descriptive statistics and corresponding log₁₀ reductions relative to the control group are presented in Table 2.

**Table 2:** Descriptive statistics and approximate log10 reductions for each experimental group CFU: colony-forming unit; CHX: chlorhexidine; SD: standard deviation

Group	n	Mean log_10_(CFU)	SD	Approx. mean CFU/implant	Approx. log_10_ reduction vs control
Control	12	5.09	0.33	155.333	Reference
CHX	12	4.03	0.12	11.125	1.06
GalvoSurge	12	3.77	0.25	6.883	1.32
Laser	12	2.52	0.39	515	2.57

Among the tested protocols, laser treatment resulted in the lowest residual bacterial burden, followed by GalvoSurge and CHX. To further assess the difference between treatment outcomes, inferential analysis focused on the comparison between CHX and laser groups, representing the least and most effective active protocols. Detailed descriptive statistics for this comparison are shown in Table 3.

**Table 3:** Descriptive statistics of log10-transformed CFU for the focused CHX versus laser comparison CFU: colony-forming unit; CHX: chlorhexidine; SD: standard deviation; SE: standard error

Compared group	n	Mean	SD	SE	95% CI lower	95% CI upper	Minimum	Maximum
CHX	12	4.0297	0.1220	0.0352	3.9522	4.1072	3.8633	4.2967
Laser	12	2.5175	0.3874	0.1118	2.2714	2.7637	2.0000	3.4150
Total	24	3.2736	0.8218	0.1678	2.9266	3.6206	2.0000	4.2967

The laser group demonstrated markedly lower mean log₁₀(CFU) values compared with CHX. The assumption of homogeneity of variance was evaluated using Levene’s test. Results indicated a violation of this assumption (p < 0.05). One-way ANOVA results for the CHX versus laser comparison showed a highly significant difference between groups (p < 0.001). The magnitude of the treatment effect was very large (η² = 0.883), indicating a substantial difference between groups.

Overall, statistical testing showed a highly significant difference in log_10_(CFU/implant) values (p < 0.001), with a very large effect size (eta squared = 0.883). Although variance homogeneity was not fully satisfied, the conclusion was supported by Welch robust testing, strengthening confidence in the observed treatment-related difference.

## Discussion

Currently, effective decontamination of contaminated implant surfaces remains challenging in peri-implantitis management, primarily due to the resistance of biofilms on micro-rough titanium surfaces. Although multiple chemical and physical approaches have been proposed, their relative efficacy remains inconsistent in the literature [11,12,20-24]. Therefore, this study compared the antimicrobial effectiveness of Er,Cr:YSGG laser irradiation, GalvoSurge electrolytic cleaning, and 0.12% CHX against *Staphylococcus aureus* biofilm under standardized in vitro conditions. Based on the findings, the null hypothesis was rejected.

All active decontamination protocols significantly reduced viable bacterial counts compared with untreated controls; however, differences in magnitude were observed. Er,Cr:YSGG laser irradiation demonstrated the greatest reduction, followed by GalvoSurge, while CHX showed the lowest antibacterial effect. This trend likely reflects the superior ability of physical methods to disrupt biofilm structure compared with chemical disinfection alone [11,12,16,22,25].

The superior performance of the Er,Cr:YSGSG laser may be attributed to its combined photomechanical and hydrokinetic effects at a wavelength of 2780 nm, which facilitate disruption of hydrated biofilm and removal of bacterial deposits from micro-rough surfaces while minimizing thermal damage under appropriate parameters [13,16,17,25]. The approximately 2.57-log reduction observed in this study supports its antimicrobial potential and is consistent with previous findings on erbium-family lasers [16,17,25,26].

GalvoSurge electrolytic cleaning also produced a marked reduction in bacterial counts. Its mechanism involves the generation of hydrogen bubbles at the implant surface, promoting detachment of biofilm and organic contaminants, particularly within implant threads and complex surface structures, through combined mechanical and electrochemical effects [27-32]. Although GalvoSurge achieved lower CFU values than CHX, the difference was not statistically significant. This may be explained by the use of a single-species biofilm model, limited sample size, and variability within groups, which may have reduced statistical power. In addition, CHX may still exhibit substantial antibacterial activity in less complex biofilm conditions, thereby reducing observable differences between treatments [20-22,33]. Clinical studies have nevertheless reported improvements in clinical parameters and radiographic bone fill when electrolytic decontamination is combined with regenerative approaches [34,35].

CHX at 0.12% demonstrated a measurable but comparatively limited antibacterial effect. Its mechanism, based on disruption of bacterial cell membranes through its cationic bisbiguanide structure, is well established [36-38]. However, its efficacy is restricted by limited penetration into structured biofilms and by the relatively low concentration used in this study [20-24,33,36]. Higher concentrations (1-2%) have been shown to produce stronger antimicrobial effects in peri-implantitis management [39,40]. Although CHX exhibits substantivity and prolonged antimicrobial activity [37,41], these properties appear insufficient for effective disruption of mature biofilm on rough implant surfaces. Additionally, its interaction with titanium surfaces has shown variable effects on wettability and residue formation, which may influence bacterial adhesion and recolonization [42,43]. The absence of a statistically significant difference between CHX and GalvoSurge should therefore be interpreted as a limitation of the experimental model rather than true equivalence in clinical efficacy.

A key strength of this study is the development and application of a standardized custom-designed 3D-printed peri-implant defect model, which allowed controlled exposure of implant threads while preserving the complex macrogeometry of the implant surface. Unlike conventional flat-surface models, this approach better reproduces the clinical challenge of biofilm contamination in peri-implant defects, where implant threads and irregular surface topography may limit access to decontamination procedures. Additionally, the study incorporated commercially available SLA titanium implants, standardized biofilm formation conditions, clinically relevant decontamination protocols, and quantitative microbiological assessment using CFU analysis, allowing direct comparison of different treatment modalities under controlled experimental conditions.

However, several limitations must be considered. The use of a single-species *Staphylococcus aureus *biofilm does not fully reproduce the complexity of peri-implantitis-associated polymicrobial biofilms, which involve diverse microbial communities, interspecies interactions, and increased resistance to antimicrobial interventions [20,21,33]. Additionally, the in vitro design does not account for important biological factors such as saliva, host immune response, tissue healing mechanisms, and functional loading conditions. CFU analysis reflects only cultivable bacterial populations and does not evaluate biofilm matrix composition, endotoxin levels, or viable-but-non-culturable microorganisms. Furthermore, this study focused primarily on antibacterial efficacy and did not assess potential alterations in implant surface morphology, chemical characteristics, or temperature changes associated with laser irradiation. The absence of surface characterization techniques, such as scanning electron microscopy and physicochemical analyses, limits conclusions regarding the preservation of implant surface integrity following decontamination procedures.

These limitations are supported by previous studies emphasizing the importance of biofilm complexity and implant surface characteristics. Khayat et al. highlighted the shift toward dysbiotic multispecies biofilms in peri-implant disease [44], while El Khoury et al. demonstrated that implant surface characteristics may influence long-term stability [45]. Similarly, Anka et al. and related histological studies underscore the need to evaluate both antibacterial efficacy and preservation of surface properties when assessing decontamination methods [46,47].

Clinically, the present findings suggest that physical decontamination methods, particularly Er,Cr:YSGG laser irradiation, may provide superior bacterial reduction compared with CHX alone. This supports the concept that effective peri-implantitis management requires disruption of biofilm structure in addition to antimicrobial action. However, translation to clinical practice should be approached cautiously. Future studies should incorporate multispecies biofilm models, assess implant surface alterations, and evaluate clinically relevant outcomes such as re-osseointegration and long-term stability. Optimization of laser parameters is also necessary to ensure effective decontamination while preserving implant surface integrity.

## Conclusions

Within the limitations of this in vitro controlled study, Er,Cr:YSGG laser irradiation produced the greatest reduction in viable *Staphylococcus aureus *counts on contaminated Straumann SLA tissue-level implant surfaces (SP WN, 4.8 mm × 10 mm). GalvoSurge electrolytic cleaning and 0.12% CHX also reduced bacterial load compared with untreated controls, with comparable reductions between these two approaches under the present experimental conditions. These findings support further investigation of laser-assisted and electrolytic decontamination strategies as potential adjunctive approaches for peri-implantitis management. However, further validation using multispecies biofilm models, different implant surface configurations, and analyses of potential surface alterations and thermal effects following treatment is required. Future clinical studies evaluating re-osseointegration potential and long-term peri-implant tissue outcomes are necessary before translating these in vitro findings into clinical recommendations.

## References

[1] Schwarz F, Derks J, Monje A, Wang HL (2018). Peri-implantitis. J Clin Periodontol.

[2] Berglundh T, Armitage G, Araujo MG (2018). Peri-implant diseases and conditions: consensus report of workgroup 4 of the 2017 World Workshop on the Classification of Periodontal and Peri-Implant Diseases and Conditions. J Periodontol.

[3] Derks J, Tomasi C (2015). Peri-implant health and disease. A systematic review of current epidemiology. J Clin Periodontol.

[4] Lee CT, Huang YW, Zhu L, Weltman R (2017). Prevalences of peri-implantitis and peri-implant mucositis: systematic review and meta-analysis. J Dent.

[5] Al-Hashedi AA, Laurenti M, Benhamou V, Tamimi F (2017). Decontamination of titanium implants using physical methods. Clin Oral Implants Res.

[6] Bollen CM, Lambrechts P, Quirynen M (1997). Comparison of surface roughness of oral hard materials to the threshold surface roughness for bacterial plaque retention: a review of the literature. Dent Mater.

[7] Quirynen M, Bollen CM (1995). The influence of surface roughness and surface-free energy on supra- and subgingival plaque formation in man. A review of the literature. J Clin Periodontol.

[8] Sanz-Martín I, Paeng K, Park H, Cha JK, Jung UW, Sanz M (2021). Significance of implant design on the efficacy of different peri-implantitis decontamination protocols. Clin Oral Investig.

[9] Louropoulou A, Slot DE, Van der Weijden F (2014). The effects of mechanical instruments on contaminated titanium dental implant surfaces: a systematic review. Clin Oral Implants Res.

[10] Mellado-Valero A, Buitrago-Vera P, Solá-Ruiz MF, Ferrer-García JC (2013). Decontamination of dental implant surface in peri-implantitis treatment: a literature review. Med Oral Patol Oral Cir Bucal.

[11] Baima G, Citterio F, Romandini M, Romano F, Mariani GM, Buduneli N, Aimetti M (2022). Surface decontamination protocols for surgical treatment of peri-implantitis: a systematic review with meta-analysis. Clin Oral Implants Res.

[12] Hart I, Wells C, Tsigarida A, Bezerra B (2024). Effectiveness of mechanical and chemical decontamination methods for the treatment of dental implant surfaces affected by peri-implantitis: a systematic review and meta-analysis. Clin Exp Dent Res.

[13] Sanders ER (2012). Aseptic laboratory techniques: plating methods. J Vis Exp.

[14] (2026). Serial dilution protocols. https://asm.org/asm/media/protocol-images/serial-dilution-protocols.pdf?ext=.pdf.

[15] (2026). Bacteriological analytical manual, chapter 3: aerobic plate count. https://www.fda.gov/media/191248/download.

[16] Kottmann L, Franzen R, Conrads G, Wolfart S, Marotti J (2023). Effect of Er,Cr:YSGG laser with a side-firing tip on decontamination of titanium disc surface: an in vitro and in vivo study. Int J Implant Dent.

[17] Karimi MR, Farkhondemehr B, Ghaeni Najafi M, Etemadi A, Chiniforush N (2021). Efficacy of titanium brush, 915 nm diode laser, citric acid for eradication of Staphylococcus aureus from implant surfaces. BMC Oral Health.

[18] Chen X, Thomsen TR, Winkler H, Xu Y (2020). Influence of biofilm growth age, media, antibiotic concentration and exposure time on Staphylococcus aureus and Pseudomonas aeruginosa biofilm removal in vitro. BMC Microbiol.

[19] Belley A, Neesham-Grenon E, McKay G (2009). Oritavancin kills stationary-phase and biofilm Staphylococcus aureus cells in vitro. Antimicrob Agents Chemother.

[20] Costerton JW, Stewart PS, Greenberg EP (1999). Bacterial biofilms: a common cause of persistent infections. Science.

[21] Mah TF, O'Toole GA (2001). Mechanisms of biofilm resistance to antimicrobial agents. Trends Microbiol.

[22] Souza JG, Lima CV, Costa Oliveira BE (2018). Dose-response effect of chlorhexidine on a multispecies oral biofilm formed on pure titanium and on a titanium-zirconium alloy. Biofouling.

[23] Brunello G, Becker K, Scotti L, Drescher D, Becker J, John G (2021). The effects of three chlorhexidine-based mouthwashes on human osteoblast-like SaOS-2 cells. An in vitro study. Int J Mol Sci.

[24] Park JB, Lee G, Yun BG, Kim CH, Ko Y (2014). Comparative effects of chlorhexidine and essential oils containing mouth rinse on stem cells cultured on a titanium surface. Mol Med Rep.

[25] Aoki A, Sasaki KM, Watanabe H, Ishikawa I (2004). Lasers in nonsurgical periodontal therapy. Periodontol 2000.

[26] Simões IG, Dos Reis AC, Valente ML (2023). Influence of surface treatment by laser irradiation on bacterial adhesion on surfaces of titanium implants and their alloys: systematic review. Saudi Dent J.

[27] Ratka C, Weigl P, Henrich D, Koch F, Schlee M, Zipprich H (2019). The effect of in vitro electrolytic cleaning on biofilm-contaminated implant surfaces. J Clin Med.

[28] Schneider S, Rudolph M, Bause V, Terfort A (2018). Electrochemical removal of biofilms from titanium dental implant surfaces. Bioelectrochemistry.

[29] Assunção MA, Botelho J, Machado V (2023). Dental implant surface decontamination and surface change of an electrolytic method versus mechanical approaches: a pilot in vitro study. J Clin Med.

[30] Park SH, Kim OJ, Chung HJ, Kim OS (2020). Effect of a Er, Cr:YSGG laser and a Er:YAG laser treatment on oral biofilm-contaminated titanium. J Appl Oral Sci.

[31] Virto L, Odeh V, Garcia-Quismondo E, Herrera D, Palma J, Tamimi F, Sanz M (2023). Electrochemical decontamination of titanium dental implants. An in vitro biofilm model study. Clin Oral Implants Res.

[32] Schlee M, Rathe F, Brodbeck U, Ratka C, Weigl P, Zipprich H (2019). Treatment of peri-implantitis-electrolytic cleaning versus mechanical and electrolytic cleaning-a randomized controlled clinical trial-six-month results. J Clin Med.

[33] Dhaliwal JS, Abd Rahman NA, Ming LC, Dhaliwal SK, Knights J, Albuquerque Junior RF (2021). Microbial biofilm decontamination on dental implant surfaces: a mini review. Front Cell Infect Microbiol.

[34] Bosshardt DD, Brodbeck UR, Rathe F, Stumpf T, Imber JC, Weigl P, Schlee M (2022). Evidence of re-osseointegration after electrolytic cleaning and regenerative therapy of peri-implantitis in humans: a case report with four implants. Clin Oral Investig.

[35] Monje A, Pons R, Peña P (2025). Electrolytic surface decontamination in the reconstructive therapy of peri-implantitis: single-center outcomes. Int J Periodontics Restorative Dent.

[36] Puisys A, Akhondi S, Vindasiute-Narbute E, Zvirblis T, Gallucci GO, Pedrinaci I (2026). Peri-implant reconstruction with autogenous bone and electrolytic therapy: a randomized controlled clinical trial. Clin Implant Dent Relat Res.

[37] Milstone AM, Passaretti CL, Perl TM (2008). Chlorhexidine: expanding the armamentarium for infection control and prevention. Clin Infect Dis.

[38] Daubert DM, Weinstein BF (2019). Biofilm as a risk factor in implant treatment. Periodontol 2000.

[39] Renvert S, Lessem J, Dahlén G, Lindahl C, Svensson M (2006). Topical minocycline microspheres versus topical chlorhexidine gel as an adjunct to mechanical debridement of incipient peri-implant infections: a randomized clinical trial. J Clin Periodontol.

[40] de Waal YC, Raghoebar GM, Meijer HJ, Winkel EG, van Winkelhoff AJ (2015). Implant decontamination with 2% chlorhexidine during surgical peri-implantitis treatment: a randomized, double-blind, controlled trial. Clin Oral Implants Res.

[41] Reda B, Hollemeyer K, Trautmann S, Volmer DA, Hannig M (2021). First insights into chlorhexidine retention in the oral cavity after application of different regimens. Clin Oral Investig.

[42] Stuani VT, Kim DM, Nagai M, Chen CY, Sant'Ana AC (2021). Effectiveness and surface changes of different decontamination protocols at smooth and minimally rough titanium surfaces. J Periodontol.

[43] Bayrak M, Kocak-Oztug NA, Gulati K, Cintan S, Cifcibasi E (2022). Influence of clinical decontamination techniques on the surface characteristics of SLA titanium implant. Nanomaterials (Basel).

[44] Khayat R, Menassa G, Chakar C (2021). Peri-implant microbiome: a literature review part II. Int Arab J Dent.

[45] El Khoury N, Gabriel M, Chakar C, Kassir AR, Naaman N (2023). Clinical and radiographic evaluation of bone remodeling around implants placed in horizontally augmented bone: a pilot study. Int Arab J Dent.

[46] Anka C, Daraze C, Faraj M, Mhanna R, Chakar C, Menassa G, Naaman N (2025). In vitro evaluation of implant surface alteration following different decontamination methods. Discov Med.

[47] Menhall A, Lahoud P, Yang KR, Park KB, Razukevicius D, Traini T, Makary C (2024). The mineral apposition rate on implants with either a sandblasted acid-etched implant surface (SLA) or a nanostructured calcium-incorporated surface (XPEED(®)): a histological split-mouth, randomized case/control human study. Materials (Basel).

